# Resin Bonding of Self-Etch Adhesives to Bovine Dentin Bleached from Pulp Chamber

**DOI:** 10.1155/2016/1313586

**Published:** 2016-09-26

**Authors:** Akiko Haruyama, Atsushi Kameyama, Junji Kato, Shinji Takemoto, Yutaka Oda, Eiji Kawada, Toshiyuki Takahashi, Masahiro Furusawa

**Affiliations:** ^1^Department of Endodontics and Clinical Cariology, Tokyo Dental College, 2-9-18 Misaki-cho, Chiyoda-ku, Tokyo 101-0061, Japan; ^2^Department of Cariology and Operative Dentistry, Division of Oral Health Sciences, Graduate School of Medical and Dental Sciences, Tokyo Medical and Dental University (TMDU), 1-5-45 Yushima, Bunkyo-ku, Tokyo 113-8549, Japan; ^3^Department of Dental Materials Science, Tokyo Dental College, 2-9-18 Misaki-cho, Chiyoda-ku, Tokyo 101-0061, Japan; ^4^Division of General Dentistry, Tokyo Dental College Chiba Hospital, 1-2-2 Masago, Mihama-ku, Chiba 261-8502, Japan

## Abstract

This study evaluated the microtensile bond strength (*μ*TBS) of 1-step self-etch adhesives (1-SEAs) and 2-step self-etch adhesives (2-SEAs) to pulp chamber dentin immediately after bleaching with 2 types of common bleaching techniques. Pulp chamber dentin of bovine teeth was bleached using 30% hydrogen peroxide (H_2_O_2_) solution with quartz-tungsten-halogen light-curing unit (Group 1) and 3.5% H_2_O_2_-containing titanium dioxide (TiO_2_) (Pyrenees®) activated with 405-nm violet diode laser for 15 min (Group 2). Unbleached specimens were placed in distilled water for 15 min and used as controls. After treatment, dentin was bonded with resin composite using 1-SEA or 2-SEA and stored in water at 37°C for 24 h. Each specimen was sectioned and trimmed to an hourglass-shape and *μ*TBS was measured. Fractured specimens were examined under a scanning electron microscope to determine fracture modes. All specimens in Group 1 failed before proper bonding tests. In Group 2, the *μ*TBS of 2-SEA was significantly greater (with no failed specimens) than 1-SEA (where 21 out of 36 failed). These results indicate that 2-SEA is a better adhesive system than 1-SEA on bleached dentin. Our results also demonstrated that application of H_2_O_2_ significantly decreases bond strength of resin to dentin; however, in the case of nonvital tooth bleaching, Pyrenees® is a better alternative to the conventional 30% H_2_O_2_ bleaching.

## 1. Introduction

Tooth bleaching techniques such as the walking bleach technique, also known as internal bleaching technique, are common treatment methods to manage esthetic concerns regarding discolored nonvital teeth caused by pulpal necrosis or by past history of root canal treatment [1, 2]. A common component of tooth bleaching agents is hydrogen peroxide (H_2_O_2_) which when catalyzed by 405-nm violet laser irradiation generates oxygen hydroxyl radicals and other species that decompose organic pigments on the teeth thereby removing dental colorants [3–6]. After bleaching nonvital teeth, endodontic access cavities are usually filled with resin composite.

The 2-step self-etch adhesive (2-SEA) system has been recognized as the “gold standard” and this system has been widely used for bonding during direct composite restoration [7]. Since the bonding agent contains acidic monomers, both the enamel and dentin can be simultaneously conditioned and primed and the etch-and-rinse phase is no longer necessary. In particular, the “mild” self-etch adhesives demineralize only the dentin to a shallow degree while leaving hydroxyapatite crystals around the collagen fibrils. This type of adhesion prevents the degradation of resin-dentin interface caused by excessive demineralization [8]. In recent years, the 1-step self-etch adhesives (1-SEAs), also known as the “all-in-one” adhesives, have become commercially available [9]. The 1-SEAs are complex mixtures with both hydrophilic and hydrophobic components containing large amounts of solvents, such as acetone, ethanol, and water. This system enables the combining of etching, priming, and bonding phase into 1 step, consequently simplifying the restoration process and reducing chair time [10].

The bonding behavior of 1-SEAs is different than that of 2-SEAs because, even after high-pressure air blow, polymerization rate and adhesive strength decrease due to their high hydrophilicity when solvent removal before light curing is not complete. As a result, the bonding behavior of 1-SEA on bleaching agent-applied dentin may also differ from that of 2-SEA. The purpose of this study was to evaluate the microtensile bond strength (*μ*TBS) of 1-SEA and 2-SEA to pulp chamber dentin immediately after bleaching with 2 types of common bleaching techniques. The null hypothesis is that there is no difference between the bonding behaviors of 1-SEA and 2-SEA on bleached dentin.

## 2. Materials and Methods

### 2.1. Tooth Preparation

Thirty bovine teeth, which were frozen after extraction to maintain freshness, were defrosted and the tooth crown was cut 3 mm below the cement-enamel junction. Each crown was sectioned mesiodistally along the long axis and the surface was ground using #400 SiC paper exposing the enamel and labial pulpal chamber dentin. The specimens were randomly divided into 3 groups of 10 teeth (Table 1).

### 2.2. Test Groups

For Group 1, pulp chamber dentin was treated with 30% H_2_O_2_ solution (Wako Pure Chemical Industries, Osaka, Japan, pH 3.35). The bleached surface was irradiated with a quartz-halogen-tungsten light-curing unit (Optilux 501, Kerr Hawe, Bioggio, Switzerland) for 15 min at a distance of 1 mm from the tip of the light source. The diameter of the irradiated area was about 8 mm and the power density was 720 mW/cm^2^.

For Group 2, pulp chamber dentin was treated with 3.5% H_2_O_2_-based bleaching agent containing TiO_2_ (Pyrenees, Mitsubishi Gas Chemical Co., Tokyo, Japan, lot number 07P0601). In order to activate the photocatalytic effect of TiO_2_, the bleached surface was irradiated with 405-nm violet diode laser (VLM 500, Sumitomo Electric Industries, Yokohama, Japan) for 15 min. The laser was delivered through an optical fiber with a core diameter of 800 *μ*m. The specimen was placed at a distance of 15 mm from the fiber tip to obtain an irradiated area of 8 mm in diameter [11, 12]. Energy levels were measured periodically with a power meter (LaserMate-P, Coherent, CA) in order to maintain irradiation at a power density of 800 mW/cm^2^.

The last group served as the control where pulp chamber dentin was placed in distilled water for 15 min.

After treatment, one specimen from each group was dehydrated and dried, placed on aluminum stab, coated with Au-Pd using an automatic sputter coater (SC500A, VG Microtech, East Sussex, UK), and surface-observed using scanning electron microscope (SEM, JSM-6340F, JEOL, Tokyo, Japan) at 15 kV.

### 2.3. Specimen Preparation for *μ*TBS

After treatment, specimens were rinsed with running tap water for 1 min and air-dried using a triple syringe. Clearfil S^3^ Bond and Clearfil SE Bond were used as 1-SEA and 2-SEA, respectively (Table 2). 1-SEA was applied on the treated dentin surface using a disposable brush for 20 s, followed by strong air-drying using a three-way syringe, and then light-cured for 10 s using quartz-tungsten-halogen light-curing unit (Optilux 501). Thereafter, resin composite (Clearfil AP-X, Kuraray Noritake Dental, shade A2) was built up incrementally in 5 steps and light-cured for 40 s each using Optilux 501 to a height of 5 mm.

2-SEA was applied under the manufacturer's instructions as in 1-SEA (cited from our previous study) [13].

**Figure 1:**
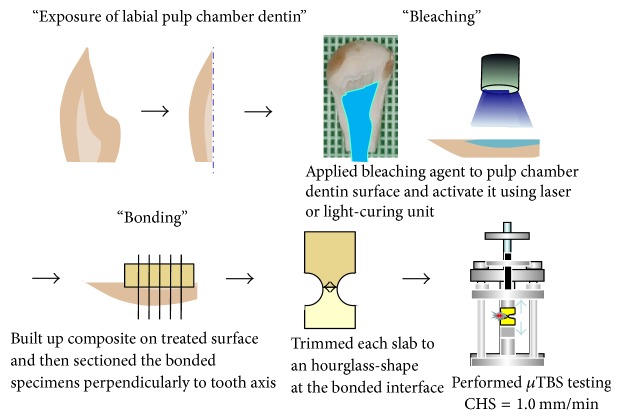
Schematic illustration of specimen preparation and *μ*TBS testing.

The bonded specimens were stored in water at 37°C for 24 h and then sectioned (0.7 mm) in a mesial-distal direction using a low-speed diamond saw (Isomet, Buehler, Lake Bluff, IL, USA). Four slabs were obtained from each tooth. The slabs were modified to an hourglass-shape at the bonded interface and standardized to produce a bonded area of 1.0 ± 0.2 mm^2^ using a superfine diamond bur (SF-114, Shofu, Kyoto, Japan) and high-speed handpiece under copious air-water spraying. Specimens were attached to Bencor Multi-T device (Danville Engineering, San Ramon, CA, USA) using cyanoacrylate glue (Model Repair II Blue, Dentsply-Sankin, Otawara, Japan) and the *μ*TBS was measured on a universal testing machine (Tensilon RTC-1150-TSD, Orientec, Tokyo, Japan) at a crosshead speed of 1.0 mm/min. After calculating the exact area of each fractured surface after measuring the dimensions with a digital caliper (CD-15 CPX, Mitutoyo, Tokyo, Japan), *μ*TBS (MPa) was measured by dividing the recorded force (N) at the time of fracture by the bond area (mm^2^) (Figure 1). If a specimen failed before proper testing, a bond strength of 0 MPa was used for statistical analyses. The number of pretesting failures was also noted.

After *μ*TBS testing, the fractured dentin-side of each specimen was placed on an aluminum stub, Au-Pd-coated, and examined under SEM (SEM: JSM-6340F, JEOL, Tokyo, Japan) to determine the mode of failure.

### 2.4. Statistical Analysis

Bonding behavior of 1-SEA and 2-SEA and bleaching techniques was compared using two-way analysis of variance (2-way ANOVA) and* post hoc* Tukey-Kramer multiple comparison test at a significance level of 5% using the IBM SPSS 18 statistical software (SPSS Inc., Chicago, IL, USA).

## 3. Results

### 3.1. SEM Observation of Treated Dentin Surface

**Figure 2:**
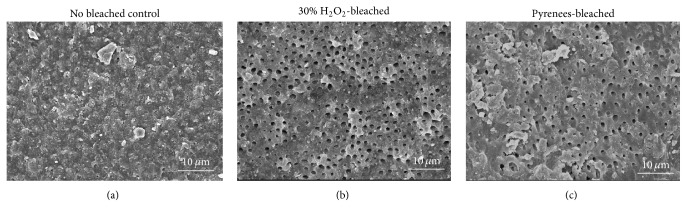
SEM images of pulp chamber dentin surface (1000x). Control: after immersion in distilled water. No dentinal tubules were exposed and the surface was entirely covered in debris. 30% H_2_O_2_-bleached surface (Group 1): dentinal tubules were exposed and no debris were detected on the dentin surface. Pyrenees-bleached surface (Group 2): dentinal tubules were not as exposed as Group 1 and some debris were present covering the tubules.

Dentinal tubules of pulpal chamber were exposed in the bleach treated groups (Figures 2(b) and 2(c)) compared to control with tubules of Group 2 being exposed to a greater degree. Dentinal surface of control specimen was covered in smear debris and dentinal plugs were present (Figure 2(a)).

### 3.2. *μ*TBS

**Figure 3:**
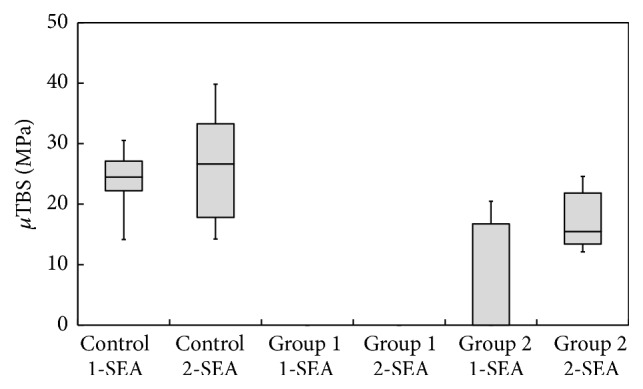
*μ*TBS of each group. The box represents the spreading of the data between the first and third quartile. The central vertical line represents the median. The whiskers denote the range of variance.

The mean *μ*TBS and SDs and the number of pretesting failures (ptf) in each group are summarized in Table 3 and graphically presented in box-whisker plots in Figure 3. The *μ*TBS of control was significantly higher than both treated groups for both adhesive systems. In the control group, no significant difference in *μ*TBS was found between 1-SEA and 2-SEA. In Group 1, all 36 specimens from both adhesive systems failed before testing. In Group 2, *μ*TBS of 2-SEA was significantly greater compared to 1-SEA. For 1-SEA, 21 out of 36 specimens failed before testing whereas no specimens failed in 2-SEA.

### 3.3. Failure Analysis

**Figure 4:**
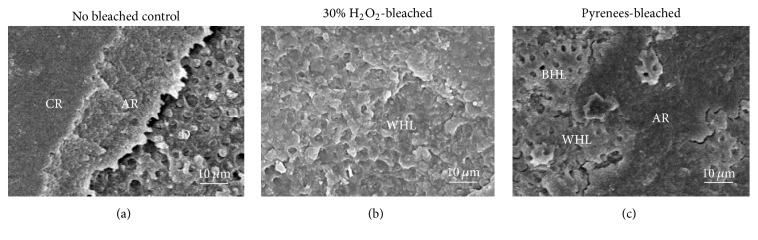
SEM images of fractured dentin-side surface of 1-SEA. (a) Control: mixture of the cohesive failures in composite resin (CR) and adhesive resin (AR) and interfacial failure between AR and dentin (D) can be observed. (b) Group 1: failure within the hybrid layer (WHL) can be seen. (c) Group 2: failure occurred within AR and within/at the bottom of the hybrid layer (WHL and BHL, resp.).

The representative SEM photomicrographs of the dentin-side of the fractured surface after *μ*TBS testing are shown in Figure 4. A mixture of cohesive failures in both dentin and composite regions was observed in a majority of cases (control, Figure 4(a)). Failures in the vicinity of adhesive interface were observed in both Group 1 and Group 2 (Figures 4(b) and 4(c), resp.) (Table 4).

## 4. Discussion

The purpose of this study was to evaluate the *μ*TBS of 1-SEA and 2-SEA to pulp chamber dentin immediately after bleaching with 2 types of bleaching techniques. No significant differences in *μ*TBS were found between 1-SEA and 2-SEA in Group 1 using 30% H_2_O_2_ whereas significant difference in *μ*TBS was found between the adhesive systems in Group 2 which used Pyrenees. From these results, the null hypothesis that there is no difference between the bonding behaviors of 1-SEA and 2-SEA on bleached dentin can be partially rejected.

In this study, *μ*TBS testing was used because it has been recognized as a suitable method for investigating resin bonding strength to pulpal dentin [14, 15]. Reports have shown that resin bond strength to H_2_O_2_-treated intracoronal dentin is lower than that to nontreated dentin [16–18]. Inhibition of resin polymerization has been reported to be one of the reasons for lower resin bond strength to bleached tooth structure [18]. In addition to the obvious negative effects of insufficient bonding, residual resin monomer at the bonded interface may cause degradation which could potentially lead to the reoccurrence of tooth discoloration. For these reasons, it is important to identify the appropriate adhesive system for optimal bonding.

There have been several reports that the bond strength of Clearfil S^3^ Bond to dentin was lower compared to Clearfil SE Bond [19, 20]. This is inconsistent with our study where there was no significant difference between *μ*TBS of 1-SEA and 2-SEA in the control group. The pH of Clearfil S^3^ Bond has been reported to be 2.7 [21], which is milder than Clearfil SE Bond; therefore, its effect on smear removal would be expected to be smaller yielding lower bonding strength. However, Ermis et al. [22] demonstrated that the *μ*TBS of Clearfil SE Bond and that of Clearfil S^3^ Bond to medium grit diamond bur-cut dentin were 60.3 ± 14.8 MPa and 8.4 ± 9.0 MPa, respectively. In comparison, the *μ*TBS to extrafine grit bur-cut dentin were 49.8 ± 18.6 MPa and 34.4 ± 22.3 MPa, respectively. In this study, the adhesive agent was applied to the pulp chamber dentin without grinding or cutting; therefore the lack of smear layer caused by grinding may have caused the *μ*TBS of 1-SEA and 2-SEA to be similar.

The *μ*TBS of 2-SEA was significantly higher than that of 1-SEA in Group 2. This can be explained in 2 reasons. Firstly, Clearfil SE Bond is well-known for its excellent bonding performance [23]. The pH of self-etching primer of Clearfil SE Bond is 1.9–2.0, which is categorized as a “mild” self-etch adhesive [21]. Instead of dissolving the smear layer, the self-etching primer of Clearfil SE Bond diffuses through the smear to produce a hybrid layer regardless of smear thickness [23]. The bonding phenomenon is a hypothesized model called the “AD concept” where MDP contained in the adhesive agent chemically bond to calcium ions decalcified from hydroxyapatite, which then copolymerize with the adhesive resin monomers [7, 24]. High filler content and high polymerization rate are responsible for the mechanical properties which contribute to its exceptional bonding performance [25–27]. Secondly, SEM images revealed high exposure of dentinal tubules in Group 1. According to studies, Clearfil S^3^ Bond is more hydrophilic than Clearfil SE Bond; therefore, a deep monomer penetration of 1-SEA may lead to difficulty removing excess solvent (water and ethanol) causing incomplete polymerization [19, 20, 28]. This explanation is also substantiated by the cohesive failures observed within the adhesive resin in the SEM images (Figure 4(a)). In Group 2, 21 out of 36 specimens failed before proper testing in 1-SEA whereas none failed when 2-SEA was used indicating that 2-SEA is the better method for bonding resin to dentin after tooth bleaching.

Two types of bleaching techniques using 30% H_2_O_2_, which is the conventional concentration used for walking bleach technique, and Pyrenees, a new photocatalytic activity technology using TiO_2_ and 3% H_2_O_2_, were used in this study. Conventional bleaching agents contain a considerable amount of hydrogen peroxide (H_2_O_2_) often causing cervical root resorption and damage to surrounding periodontal tissue [29, 30]. The addition of TiO_2_ to bleaching agents followed by activation using a violet light source enhances the reaction of hydrogen peroxide (H_2_O_2_) in the bleaching agent enabling the reduction of H_2_O_2_ from 30–35% to 3.5% [4]. As a result, serious side effects of residual oxygen may be reduced leading to fewer cases of cervical root resorption and periodontal damage [31].

An important finding was that all the specimens treated with H_2_O_2_-containing bleaching systems had significantly lower *μ*TBS compared to the control. A study suggests that the reduction in bond strength is due to the decreased mechanical strength of dentin as a result of the oxidizing effect of peroxide [32–34]. Our SEM results, however, did not support this hypothesis because no failure in dentin was observed. However, our results for reduced bonding of resin to H_2_O_2_ treated dentin can be explained by residual oxygen remaining in dentin pores after bleaching, which inhibits resin polymerization cured through the free radical mechanism [4]. In addition, the high acidity of hydrogen peroxide could have excessively demineralized the dentin surface affecting bonding strength [18].

In this study, not only did the specimens treated with conventional 30% H_2_O_2_ fail before proper testing, but Pyrenees treated groups showed significantly higher *μ*TBS compared to the conventional H_2_O_2_ treated group. The bleaching effect of Pyrenees has been reported to be equal to the walking bleach technique using 30–35% H_2_O_2_ and sodium perborate [35, 36]. This indicates that bleaching agent containing TiO_2_ is a better alternative to the traditional walking bleach technique from the viewpoint of safety and adhesive dentistry.

In this study, resin was bonded to dentin immediately after bleaching; however, delayed bonding and the application of antioxidants on treated surfaces to reverse the damage of H_2_O_2_ on the dentin surface are areas of further interest when considering the situation in a clinical setting [37].

## 5. Conclusion

This study evaluated the *μ*TBS of 1-SEA and 2-SEA to pulp chamber dentin immediately after bleaching with 2 types of common bleaching techniques. In the 30% H_2_O_2_ treated groups, all specimens failed before proper bonding tests. In the Pyrenees treated group, the *μ*TBS of 2-SEA was significantly greater, with no failed specimens, than 1-SEA where 21 out of 36 specimens failed. These results therefore indicate that 2-SEA is a better adhesive system than 1-SEA on bleached dentin. Our results also demonstrated that application of H_2_O_2_ significantly decreases bonding strength of resin to dentin; however, in the case of bleaching nonvital tooth, Pyrenees is a better alternative to the conventional 30% H_2_O_2_ bleaching agent.

## Figures and Tables

**Table 1:** Test groups.

	Agent	Light activation
Control	Water	—
Group 1	30% H_2_O_2_	Optilux 501
Group 2	3.5% H_2_O_2_ + TiO_2_	405-nm diode laser

**Table 2:** Dental adhesives used in this study.

Code	Product	Components	pH	Application protocol
1-SEA	Clearfil S^3^ Bond Kuraray Noritake Dental, Kurashiki, Japan	10-MDP, Bis-GMA, HEMA, initiator ethanol, water, stabilizer, filler, hydrophobic dimethacrylate	2.7	(1) Apply adhesive for 20 s (2) Relatively strong stream of air for drying (3) Light cure for 10 s

2-SEA	Clearfil SE Bond Kuraray Noritake Dental, Kurashiki, Japan	Primer: 10-MDP, HEMA, hydrophilic DMA, photoinitiator, aromatic tertiary amine, water Bonding: 10-MDP, Bis-GMA, HEMA, hydrophobic DMA, photoinitiator, aromatic tertiary amine, silanized colloidal silica	1.9	(1) Apply primer for 20 s (2) Gently air-drying (3) Apply bonding agent (4) Light cure for 20 s

**Table 3:** Microtensile bond strength (mean ± SD, MPa) and the number of the pretesting failures (*n* = 36).

	1-SEA	2-SEA^**∗**^
Control	24.0 ± 5.6^a^ (0)	26.5 ± 9.8^a^ (0)
Group 1	0.0 ± 0.0^d^ (36)	0.0 ± 0.0^d^ (36)
Group 2	7.6 ± 9.4^c^ (21)	17.3 ± 5.8^b^ (0)

The same superscript letters represent no statistical differences (Tukey-Kramer test; *p* > 0.05).

^*∗*^ *μ*TBS values of 2-SEA were cited from Haruyama et al., 2010 [13].

**Table 4:** Failure patterns in *µ*TBS specimens.

	Interfacial^a^	Dentin^b^	Resin^c^	Mixed^d^	Total
Control					
1-SEA	7	0	0	29	36
2-SEA	9	16	0	11	36
Group 1					
1-SEA	36	0	0	0	36
2-SEA	36	0	0	0	36
Group 2					
1-SEA	22	0	2	12	36
2-SEA	26	2	6	2	36

^a^Failure in the adhesive interface and/or failure within the hybrid layer.

^b^Cohesive failure mainly within the dentin.

^c^Cohesive failure mainly within the resin.

^d^Mixture of interfacial and cohesive failures.

## References

[1] Nutting E. B., Poe G. S. (1967). Chemical bleaching of discolored endodontically treated teeth. *Dental Clinics of North America*.

[2] Anitua E., Zabalegui B., Gil J., Gascon F. (1990). Internal bleaching of severe tetracycline discolorations: four-year clinical evaluation. *Quintessence International*.

[3] Nakazawa T., Kato J., Akashi G., Igarashi A., Hirai Y. (2007). Effect of tooth bleaching on low concentration hydrogen peroxide containing titanium dioxide photocatalyst. *The Japanese Journal of Conservative Dentistry*.

[4] Sakai K., Kato J., Kurata H. (2007). The amounts of hydroxyl radicals generated by titanium dioxide and 3.5% hydrogen peroxide under 405-nm diode laser irradiation. *Laser Physics*.

[5] Suemori T., Kato J., Nakazawa T., Akashi G., Hirai Y. (2008). A new non-vital tooth bleaching method using titanium dioxide and 3.5% hydrogen peroxide with a 405-nm diode laser or a halogen lamp. *Laser Physics Letters*.

[6] Suyama Y., Otsuki M., Ogisu S. (2009). Effects of light sources and visible light-activated titanium dioxide photocatalyst on bleaching. *Dental Materials Journal*.

[7] Van Meerbeek B., Yoshihara K., Yoshida Y., Mine A., De Munck J., Van Landuyt K. L. (2011). State of the art of self-etch adhesives. *Dental Materials*.

[8] De Munck J., Van Meerbeek B. (2007). The current status of bonding to dentin anno 2007. *International Journal of Oral-Medical Sciences*.

[9] Perdigão J. (2007). New developments in dental adhesion. *Dental Clinics of North America*.

[10] De Munck J., Van Meerbeek B., Vargas M. (2005). One day bonding effectiveness of new self-etch adhesives to bur-cut enamel and dentin. *Operative Dentistry*.

[11] Kotoku Y., Kato J., Akashi G., Hirai Y., Ishihara K. (2009). Bactericidal effect of a 405-nm diode laser on *Porphyromonas gingivalis*. *Laser Physics Letters*.

[12] Kameyama A., Hatayama H., Kato J. (2011). Light-curing of dental resins with GaN violet laser diode: the effect of photoinitiator on mechanical strength. *Lasers in Medical Science*.

[13] Haruyama A., Kato J., Kameyama A., Hirai Y., Oda Y. (2010). Effect of titanium dioxide and 3.5% hydrogen peroxide with 405-nm diode laser irradiation on bonding of resin to pulp chamber dentin. *Laser Physics*.

[14] Sano H., Shono T., Sonoda H. (1994). Relationship between surface area for adhesion and tensile bond strength—evaluation of a micro-tensile bond test. *Dental Materials*.

[15] Lopes G. C., De Carvalho Cardoso P., Vieira L. C. C., Baratieri L. N. (2004). Microtensile bond strength to root canal vs pulp chamber dentin: effect of bonding strategies. *Journal of Adhesive Dentistry*.

[16] Demarco F. F., Turbino M. L., Jorge A. G., Matson E. (1998). Influence of bleaching on dentin bond strength. *American Journal of Dentistry*.

[17] Elkhatib H., Nakajima M., Hiraishi N., Kitasako Y., Tagami J., Nomura S. (2003). Surface pH and bond strength of a self-etching primer/adhesive system to intracoronal dentin after application of hydrogen peroxide bleach with sodium perborate. *Operative Dentistry*.

[18] Nomoto S., Kameyama A., Nakazawa T. (2006). Influence of ascorbic acid on bonding of peroxide-affected dentin and 4-META/MMA-TBB resin. *Clinical Oral Investigations*.

[19] Kameyama A., Aizawa K., Kato J., Hirai Y. (2009). Tensile bond strength of single-step self-etch adhesives to Er:YAG laser-irradiated dentin. *Photomedicine and Laser Surgery*.

[20] Van Landuyt K. L., Mine A., De Munck J. (2009). Are one-step adhesives easier to use and better performing? Multifactorial assessment of contemporary one-step self-etching adhesives. *The Journal of Adhesive Dentistry*.

[21] Koshiro K., Sidhu S. K., Inoue S., Ikeda T., Sano H. (2006). New concept of resin-dentin interfacial adhesion: the nanointeraction zone. *Journal of Biomedical Materials Research Part B: Applied Biomaterials*.

[22] Ermis R. B., De Munck J., Cardoso M. V. (2008). Bond strength of self-etch adhesives to dentin prepared with three different diamond burs. *Dental Materials*.

[23] Tay F. R., Pashley D. H. (2001). Aggressiveness of contemporary self-etching systems. I. Depth of penetration beyond dentin smear layers. *Dental Materials*.

[24] Yoshida Y., Nagakane K., Fukuda R. (2004). Comparative study on adhesive performance of functional monomers. *Journal of Dental Research*.

[25] Cadenaro M., Antoniolli F., Sauro S. (2005). Degree of conversion and permeability of dental adhesives. *European Journal of Oral Sciences*.

[26] Kameyama A., Kato J., Yoshinari M., Kotoku Y., Akashi G., Hirai Y. (2008). Ultimate micro-tensile strength of dental adhesives cured at different light source. *Journal of Photopolymer Science and Technology*.

[27] Kameyama A., Kato J., De Munck J. (2011). Light-curing efficiency of dental adhesives by gallium nitride violet-laser diode determined in terms of ultimate micro-tensile strength. *Bio-Medical Materials and Engineering*.

[28] Abo H., Kameyama A., Haruyama A. (2016). Clinical observation of the tooth surface during air-drying of self-etching primer under 3D video microscope. *Applied Adhesion Science*.

[29] Harrington G. W., Natkin E. (1979). External resorption associated with bleaching of pulpless teeth. *Journal of Endodontics*.

[30] Trope M. (1997). Cervical root resorption. *Journal of the American Dental Association*.

[31] Dietschi D. (2006). Nonvital bleaching: general considerations and report of two failure cases. *The European Journal of Esthetic Dentistry*.

[32] Torneck C. D., Titley K. C., Smith D. C., Adibfar A. (1990). Adhesion of light-cured composite resin to bleached and unbleached bovine dentin. *Endodontics & Dental Traumatology*.

[33] Lewinstein I., Hirschfeld Z., Stabholz A., Rotstein I. (1994). Effect of hydrogen peroxide and sodium perborate on the microhardness of human enamel and dentin. *Journal of Endodontics*.

[34] Tam L. E., Noroozi A. (2007). Effects of direct and indirect bleach on dentin fracture toughness. *Journal of Dental Research*.

[35] Nakazawa T., Kato J., Suemori T. (2008). Non-vital bleach method using low-concentration hydrogen peroxide with titanium dioxide. *The Japanese Journal of Conservative Dentistry*.

[36] Nonami T., Ishibashi K., Ishibashi T., Kondo O. (2001). Bleaching of TiO_2_ photocatalyst: part 1. Color alteration and microstructural changes by bleaching. *The Japanese Journal of Conservative Dentistry*.

[37] Whang H. H., Shin D. H. (2015). Effects of applying antioxidants on bond strength of bleached bovine dentin. *Restorative Dentistry & Endodontics*.

